# *Clostridium butyricum* Strain CCFM1299 Reduces Obesity via Increasing Energy Expenditure and Modulating Host Bile Acid Metabolism

**DOI:** 10.3390/nu15204339

**Published:** 2023-10-11

**Authors:** Jingyi Liao, Yaoliang Liu, Ye Yao, Jie Zhang, Hongchao Wang, Jianxin Zhao, Wei Chen, Wenwei Lu

**Affiliations:** 1 State Key Laboratory of Food Science and Resources, Jiangnan University, Wuxi 214122, China; 18352515896@163.com (J.L.); liuyaoliang0606@163.com (Y.L.); yaoyejn@126.com (Y.Y.); jiezhang@stu.jiangnan.edu.cn (J.Z.); hcwang@jiangnan.edu.cn (H.W.); jxzhao@jiangnan.edu.cn (J.Z.); chenwei66@jiangnan.edu.cn (W.C.); 2 School of Food Science and Technology, Jiangnan University, Wuxi 214122, China; 3 National Engineering Research Center for Functional Food, Jiangnan University, Wuxi 214122, China; 4 (Yangzhou) Institute of Food Biotechnology, Jiangnan University, Yangzhou 225004, China

**Keywords:** *Clostridium butyricum* CCFM1299, obesity, energy expenditure, thermogenesis, immune-related genes, bile acids

## Abstract

*Clostridium butyricum* is a butyrate-producing microorganism which has beneficial effects on various diseases, including obesity. In our previous study, the anti-obesity *Clostridium butyricum* strain CCFM1299 (C20_1_1) was selected, but its anti-obesity mechanism was not clarified. Herein, CCFM1299 was orally administrated to high-fat-diet-treated C57BL/6J mice for 12 weeks to uncover the way the strain alleviates obesity. The results indicated that CCFM1299 alleviated obesity through increasing the energy expenditure and increasing the expression of genes related to thermogenesis in brown adipose tissue (BAT). Moreover, strain CCFM1299 could also affect the expression of immune-related genes in epididymal white adipose tissue (eWAT). This immunomodulatory effect might be achieved through its influence on the complement system, as the expression of the complement factor D (CFD) gene decreased significantly. From the view of metabolites, CCFM1299 administration increased the levels of ursodeoxycholic acid (UDCA) in feces and taurohyodeoxycholic acid (THDCA) in serum. Together, the anti-obesity potential of CCFM1299 might be attributed to the increase in energy consumption, the regulation of immune-related gene expression in eWAT, and the alteration of bile acid metabolism in the host. These provided new insights into the potential application of anti-obesity microbial preparations and postbiotics.

## 1. Introduction

Obesity is a multifactorial disease with a range of metabolic disorders. It has already become an epidemic in some developed countries, which has seriously increased national economic burdens. Obesity will not only lead to poor health, but also is the main cause of death and increases the risk of type 2 diabetes (T2D), nonalcoholic fatty liver disease (NAFLD), cancer, and other diseases [1,2]. Studies have pointed out that weight management can help diabetic patients control their blood glucose levels [3], and losing weight is an effective way to prevent obesity-related cancer [4]. Undeniably, the relationship between obesity-related diseases and obesity per se is the key to treating these diseases [2], and weight management has become the therapeutic goal of many obesity-related diseases. Therefore, it is very important to find effective and safe methods to prevent and treat obesity.

Traditional methods of losing weight mainly include bariatric surgeries, diet intervention, exercise, and pharmacotherapy. Although bariatric surgeries can effectively reduce weight, some serious mental health problems may be produced after operation [5]. Dietary intervention and exercise have the disadvantage of poor compliance, while pharmacotherapy for obesity always causes side effects, such as adverse gastrointestinal reactions [6]. As the research progresses, the role of microbiota in the development of obesity has been continuously revealed. Obesity will lead to changes in microbiota, which in turn will affect the metabolism of the host by regulating intestinal physiology, appetite, and tissue inflammation [7]. In other words, microbiota has become a therapeutic target of obesity.

Microbial preparation can prevent and treat obesity by targeting microbiota. Most of these microorganisms are intestinal symbionts with anti-obesity potential. It can reduce obesity through (a) reducing appetite and energy intake [8,9]; (b) affecting the process of intestinal digestion and absorption and balancing the energy metabolism [10]; (c) inhibiting tissue inflammation and maintaining the function of tissues [11]; and (d) improving metabolism and promoting thermogenesis of brown adipose tissue (BAT) [12]. The beneficial effects of these intestinal symbionts can also be achieved by participating in the metabolism of intestinal substances, which leads to the alteration of the makeup of luminal metabolites. Those metabolites can act as the available energy source of the host [13], and they can also serve as the signal molecules to modulate the function and immune homeostasis of intestinal or extraintestinal tissues [14]. Bile acid is the main component of bile, and luminal microorganisms can affect the bile acid spectrum through metabolizing primary bile acids into a range of secondary bile acids. Taurine-conjugated cholic acid (TCA) and deoxycholic acid (DCA) have been proved to be able to reduce obesity through promoting thermogenesis [15], while ursodeoxycholic acid (UDCA) amends hyperglycemia and intestinal barrier damage under the pathological condition of obesity [16].

As one of the intestinal symbionts, *Clostridium butyricum* has been reported to have the potential to alleviate obesity [17,18], but its mechanism of relieving obesity has not been widely revealed. *Clostridium butyricum* strain CCFM1299 (original number C20_1_1), which can effectively resist obesity, was screened out in our previous study [19], and this study aims to clarify the relationship between this strain and obesity, in which the possible ways that CCFM1299 reduces obesity and its influence on host metabolites will be explored. Herein, we found that strain CCFM1299 can alleviate the metabolic disorders of obesity, promote the transcription of thermogenic genes in brown adipose tissue (BAT), and affect the expression of immune-related genes in epididymal white adipose tissue (eWAT) under a high-fat diet (HFD). In terms of metabolites, the concentrations of UDCA in feces and THDCA in serum were significantly increased with the intervention of CCFM1299. In general, this study revealed the way for CCFM1299 to relieve obesity, pointed out that this strain could influence the bile acid metabolism of the host, and provided new insights for *Clostridium butyricum* to alleviate obesity.

## 2. Materials and Methods

### 2.1. Preparation of CCFM1299 Strain

*Clostridium butyricum* strain CCFM1299 (originally number C20_1_1) was isolated from human feces and preserved in Jiangnan University Culture Collection of Food Microbiologies (Wuxi, China). In brief, the strain was cultured anaerobically with Reinforced Clostridial Medium (RCM) at 37 °C. At the end of logarithmic phase, the bacterial suspension was centrifuged, and the bacterial precipitate was resuspended with saline containing 0.05% L- cysteine hydrochloride to ensure the concentration of bacterial suspension reached 5 × 10^8^ CFU/mL. The specific methods for the culture of this strain have been mentioned in our previous research [19].

### 2.2. Animal Experiments

Five-week-old male C57BL/6J mice (specific-pathogen-free (SPF), 19–20 g) purchased from Charles River Laboratory Animal Technology Co., Ltd., (Jiaxing, China) were kept in SPF-grade animal facility (temperature 23 ± 2 °C, humidity 60 ± 20%, light/dark period 12 h). All experimental mice were randomly divided into five groups (normal chow (NC) group; high-fat diet (HFD) group; CCFM1299 group; orlistat group; and DMSO group), and each group contained eight mice (*n* = 8). After the mice adapted to the environment of the facility for 1 week, the 12-week experiment was initiated. The mice in the NC group were fed with normal chow diet, while the mice in the other five groups were fed with HFD. The energy supply of these two feeds was consistent with the previous study, and all feeds were purchased from TROPHIC Animal Feed High (Nantong, Jiangsu). During the 12-week intervention, the mice in the orlistat group were orally given 0.2 mL orlistat suspension at 50 mg/kg once a day (orlistat powder was dissolved in 1% DMSO solution via ultrasound), and the mice in the DMSO group were given 0.2 mL of 1% DMSO solution as control. The mice in the CCFM1299 group orally received 0.2 mL bacterial suspension (the density was 5 × 10^8^ CFU/mL), while all mice in NC group and HFD group were given 0.2 mL of saline as control. At the eleventh week of intervention, the metabolism of mice was measured with a comprehensive monitoring system.

### 2.3. Adipose Tissue Histology

After the sacrifice of mice, inguinal white adipose tissue (iWAT), eWAT, and BAT were immersed in 4% paraformaldehyde for 24 h. Then, tissue blocks were made into sections through dehydration, immersion in wax, embedding, and staining with hematoxylin and eosin (H&E). A digital slice scanner (pannoramic midi ii, 3dhistch, Budapest, Hungary) was used to scan the sections.

### 2.4. Detection of Serum Biochemical Indicators

After the blood of the mice was kept at room temperature for 2 h, it was centrifuged at 3000 rpm for 15 min, and the supernatant (serum) was carefully sucked into an enzyme-free centrifuge tube. A total of 100 µL serum was diluted to 300 µL with saline, which was then used for the detection of high-density lipoprotein cholesterol (HDL-C), low-density lipoprotein cholesterol (LDL-C), total triglyceride (TG), total cholesterol (TC), fasting blood glucose (FBG), alanine aminotransferase (ALT), and aspartate aminotransferase (AST). Beckman au5800 automatic biochemical analyzer (Brea, CA, USA) was used to detect the above indicators.

### 2.5. RNA Extraction from Tissues and Quantitative PCR (qPCR)

The BAT and eWAT tissues were preserved in the RNA keeper at −80 °C. An extraction method using TRIzol, isopropanol, and chloroform was performed to isolate the total RNA from BAT and eWAT tissue. An All-in-one RT SuperMix (Vazyme, Wuhan, Hubei) was used for the reverse transcription of RNA from BAT. Quantitative PCR (qPCR) using SYBR qPCR Master Mix (Vazyme, Wuhan, Hubei) was performed on a CFX96 Touch Real-Time PCR Detection System (Bio-Rad, CA, USA) to detect the transcription level of *Ucp1*, *Pparγ*, *Pgc1α*, and *Prdm16* in BAT, and the primers used were as follows: Ucp1F, 5′-CACGGGGACCTACAATGCTT-3′; Ucp1R, 5′-ACAGTAAATGGCAGGGGACG-3′; PparγF, 5′-AAGAAGCGGTGAACCACTGA-3′; PparγR, 5′-GGAATGCGAGTGGTCTTCCA-3′; Pgc1αF, 5′-CAGCTGCCTTATTGGTTTCGTT-3′; Pgc1αR, 5′-AGCAGCACACTGGTTGGAAG-3′; Prdm16F, 5′-TCCCACCAGACTTCGAGCTA-3′; Prdm16R, 5′-CAAAGTCGGCCTCCTTCAGT-3′; βactin, 5′-TGAGCTGCGTTTTACACCCT-3′; βactin, 5′-GCCTTCACCGTTCCAGTTTT-3′.

### 2.6. Transcriptome Analysis

The RNA isolated from eWAT was used for whole transcriptome sequencing and analysis. The quality of raw data was evaluated with FastQC. The sequences in Fastq files were compared with the mouse reference genome using RNA STAR (version 2.7.0e). Subsequently, Samtools was used to sort the sequences, and the format of files was converted into BAM files. Featurecounts were used to calculate the counts of gene expression in BAM files. EdgeR (version 3.7) was used to calculate the value of transcripts read per thousand bases per million mapping (TPM). Genes satisfying log_2_ (fold change) ≥1.5 or ≤−1.5 and *p* values < 0.05 were regarded as differential genes (DGs), which were then used for KEGG enrichment analysis and GO ontology analysis. RNA-seq data were published on NCBI and the accession number is PRJNAA992647.

### 2.7. Untargeted Metabonomics

The fecal samples were quenched in liquid nitrogen and then preserved at −80 °C. The process of metabolite extraction and detection was as follows: 500 µL extractive solution (methanol, acetonitrile, and ultrapure water were mixed at a volume ratio of 2:2:1) was added into the fecal samples, which were then homogenized (35 Hz, 4 min) and ultrasonically processed (5 min, in ice-water bath) three times. The homogeneous slurries were placed at −40 °C for 1 h to precipitate impurities such as protein, and then they were centrifuged for 15 min (12,000 rpm, 4 °C). The supernatants were filtered to form the samples used for the detection. We took the same volume of all samples and mixed them to form the quality control (QC) sample. Vanquish ultra-performance liquid chromatography (Thermo Fisher Scientific, Waltham, MA, USA) equipped with Waters ACQUITY UPLC BEH Amide (2.1 mm × 100 mm, 1.7 µm) was used to separate and detect the compounds in the samples. The mobile phase consisted of ultrapure water containing 25 mmol/L ammonium acetate and 25 mmol/L ammonia water (phase A) and acetonitrile (phase B). Sample tray temperature was 4 °C and the injection volume was 2 µL. The original data file was converted into mzXML format with ProteoWizard software, and the peak identification, peak extraction, peak arrangement, and integration were carried out with R package XCMS (v 3.2). In total, 15,374 peaks extracted from the original data were pretreated (deviation filtering, missing value filtering, missing value filling, and data normalization), and 12,400 peaks were retained. After the peak was annotated with MS2 database and HDMB, the data were normalized. The logarithmization of data was performed using SIMCA software (v 16.0.2), and OPLS-DA modeling analysis was conducted, in which the VIP value was calculated. T test (Student’s *t* test) was used to calculate *p* values, and Fold change (FC) was the quantitative ratio of substances. VIP > 1, *p* values < 0.05, and FC > 1.5 or FC < 0.5 were used as standards to screen the different substances.

### 2.8. Detection of Bile Acids in Serum

The process of extraction and detection of bile acid metabolites in serum was as follows: an equal volume of extraction solution (methanol and acetonitrile were mixed at same volume) was added into the serum samples, then these mixtures were vortexed for 30 s and ultrasonically processed in ice-water bath for 10 min. After being placed at −40 °C for 1 h, the mixtures were centrifuged at 12,000 rpm for 15 min under 4 °C. The supernatants were isolated in the injection bottle. The bile acid standards were weighed and we made them into 1 mg/mL solutions, respectively. The above standard solutions were mixed at certain volumes, and this mixture was diluted in gradients to form the standard mixtures with different concentrations, which were used to draw a standard curve. Vanquish ultra-high performance liquid chromatography with Waters ACQUITY UPLC BEH C18 column (150 × 2.1 mm, 1.7 µm, Waters) was used for separation and detection of the bile acids. The mobile phase A was 5 mmol/L ammonium acetate in ultrapure water, and the mobile phase B was acetonitrile. The column temperature was set at 45 °C, and the injection volume was 1 µL. The temperature of sample tray was set to 4 °C. After filtering the original data and recording missing values, the bile acid metabolites in the sample were quantified using the standard curve. Student’s t-test was used to calculate the significant differences in the concentration of bile acid metabolites between HFD group and CCFM1299 group. *p* values < 0.05 indicated that the concentration of bile acid substances was significantly different between these two groups.

### 2.9. Statistical Analysis

Graphpad Prism (v. 8.4.3) was used for statistical analysis of body weight, eWAT index, iWAT index and BAT index, serum biochemical indicators, respiratory exchange ratio (RER), O_2_ consumption, CO_2_ production, the motor activity, and the expression level of genes in BAT. The experimental data were expressed by mean ± standard error of the mean (SEM). The significant differences between groups were calculated using one-way ANOVA with Dunnett’s multiple comparison test or Student’s *t*-test. The threshold of statistical significance was set to *p* values < 0.05.

## 3. Results

### 3.1. CCFM1299 Reduced Weight Gain and Fat Accumulation

**Figure 1:**
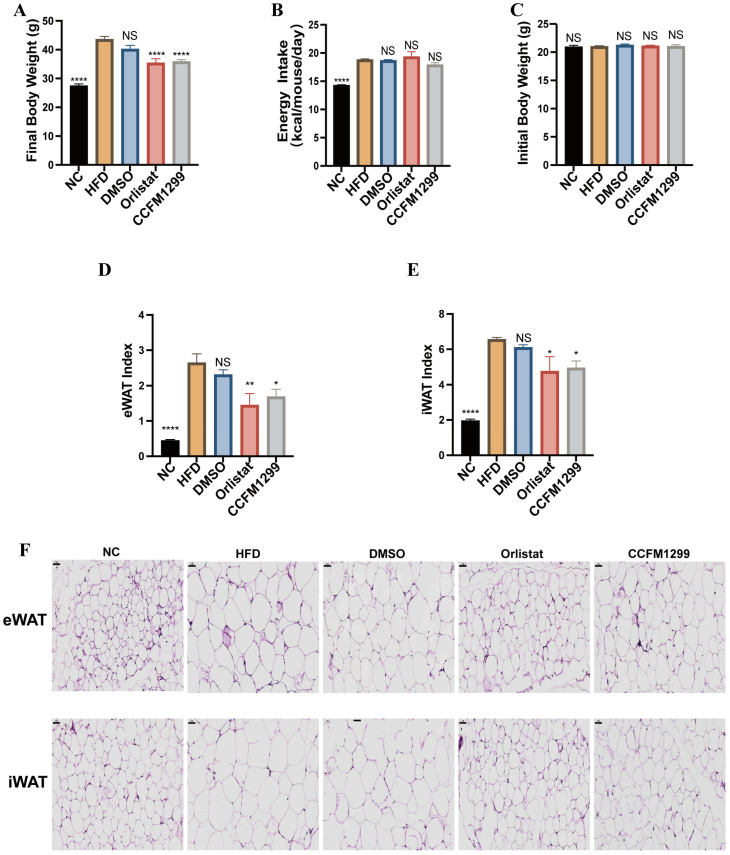
Effects of CCFM1299 and orlistat on body weight and adiposity: (**A**) body weight of each group in last week; (**B**) energy intake of each group; (**C**) initial body weight of each group; (**D**) eWAT index of mice in each group; (**E**) iWAT index of mice in each group; (**F**) representative histology of eWAT and iWAT (scale bars: 50 µm; magnification: 200×). Data are shown as means with standard error of the mean (SEM); NS was used to indicate that there was no significant difference with HFD group. *, **, and **** were used to indicate that the *p* values were less than 0.05, 0.01, and 0.0001, respectively, compared with the HFD group. One-way ANOVA followed by Dunnett’s test for multiple comparison was used for significance analysis.

Taking orlistat as a positive control, the inhibitory effects of strain CCFM1299 on body weight gain and fat accumulation were explored in this study. Compared with the NC group, the weight of mice in the HFD group increased by 58.8% under the HFD, while strain CCFM1299 and orlistat both significantly slowed the weight gain without altering the energy intake of individual mice (Figure 1A,B). There was no difference in the initial body weight of each group (Figure 1C). In order to further explore whether the strain CCFM1299 affected fat accumulation in mice, we compared the eWAT index and iWAT index (adipose tissue index is the ratio of adipose tissue weight to corresponding body weight of mice) in each group. Similar to body weight, CCFM1299 and orlistat both efficiently inhibited the increase in the iWAT and eWAT indexes (Figure 1D,E), and the size of adipocytes in eWAT and iWAT also decreased (Figure 1F). The above results showed that CCFM1299 could obviously alleviate weight gain and fat accumulation in mice, which was similar to the effect of orlistat.

### 3.2. CCFM1299 Alleviated the Hyperglycemia, Dyslipidemia, and Liver Damage Caused by HFD

**Figure 2:**
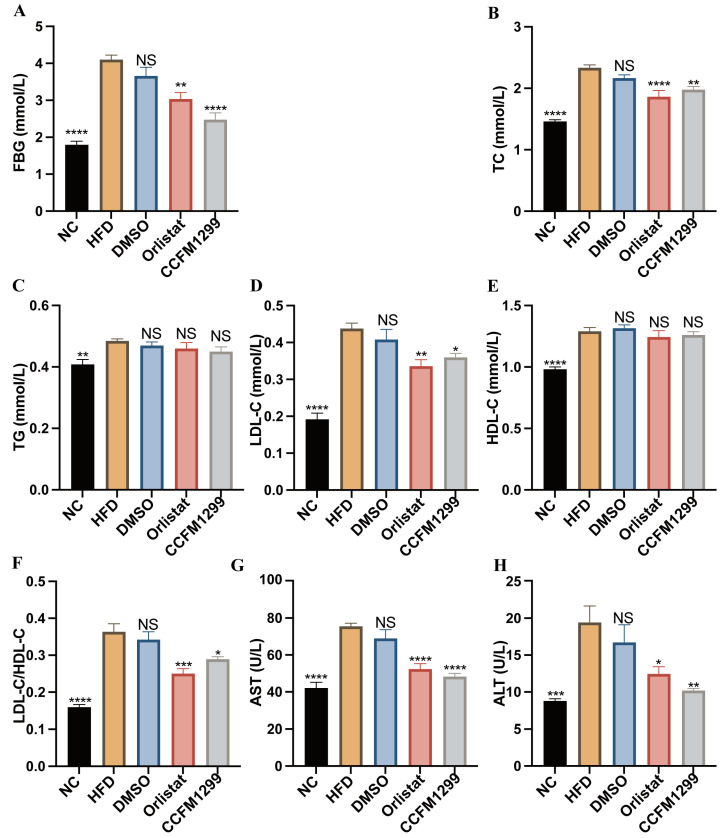
Effects of CCFM1299 and orlistat on serum lipid and glucose: (**A**) serum levels of fasted blood glucose; (**B**) serum levels of TC; (**C**) serum levels of TG; (**D**) serum levels of LDL-C; (**E**) serum levels of HDL-C; (**F**) ratio of LDL-C/HDL-C; (**G**) serum levels of AST; (**H**) serum levels of ALT. Data are shown as means with standard error of the mean (SEM); NS was used to indicate that there was no significant difference with the HFD group. *, **, ***, and **** were used to indicate that the *p* values were less than 0.05, 0.01, 0.001, and 0.0001, respectively, compared with the HFD group. One-way ANOVA followed by Dunnett’s test for multiple comparison was used for significance analysis.

Excessive weight gain and fat accumulation often results in the abnormality of blood glucose and lipid. In order to evaluate the effects of strain CCFM1299 and orlistat on glucose and lipid metabolism in mice, we measured the levels of FBG, TC, TG, LDL-C, and HDL-C in serum. Compared with the HFD group, CCFM1299 and orlistat interventions both were able to act against the increase in FBG, TC, and LDL-C in serum, but they did not effectively reverse the alterations in serum TG and HDL-C (Figure 2A–E). Interestingly, the level of serum HDL-C in the HFD group was higher than that in the NC group (Figure 2E). Studies have found that HDL-C is negatively correlated with the risk of metabolic diseases such as obesity, and is beneficial to host metabolism. However, with the deepening of research, some studies have found that excessive HDL-C in serum promotes the pathogenesis of cardiovascular and metabolic diseases [20]. Therefore, the relationship between HDL-C level and diseases is still controversial. The ratio of LDL-C to HDL-C (LDL-C/HDL-C) could reflect the lipid transport in vivo, and this ratio decreased significantly under the intervention of strains CCFM1299 and orlistat (Figure 2F).

The disorder of glucose and lipid metabolism could trigger the intrahepatic accumulation of lipid, which is related to the impairment of liver function and causes hepatic diseases, such as NAFLD. The serum level of ALT and AST could reflect the degree of liver damage. The increased serum levels of ALT and AST were found in mice in the HFD group, while strains CCFM1299 and orlistat effectively reduced the serum levels of these two transaminases (Figure 2G,H). Overall, the effects of strain CCFM1299 on blood glucose, blood lipid, and transaminases were consistent with our previous research.

### 3.3. CCFM1299 Promoted the Energy Expenditure and the Expression of Thermogenic Genes in BAT

**Figure 3:**
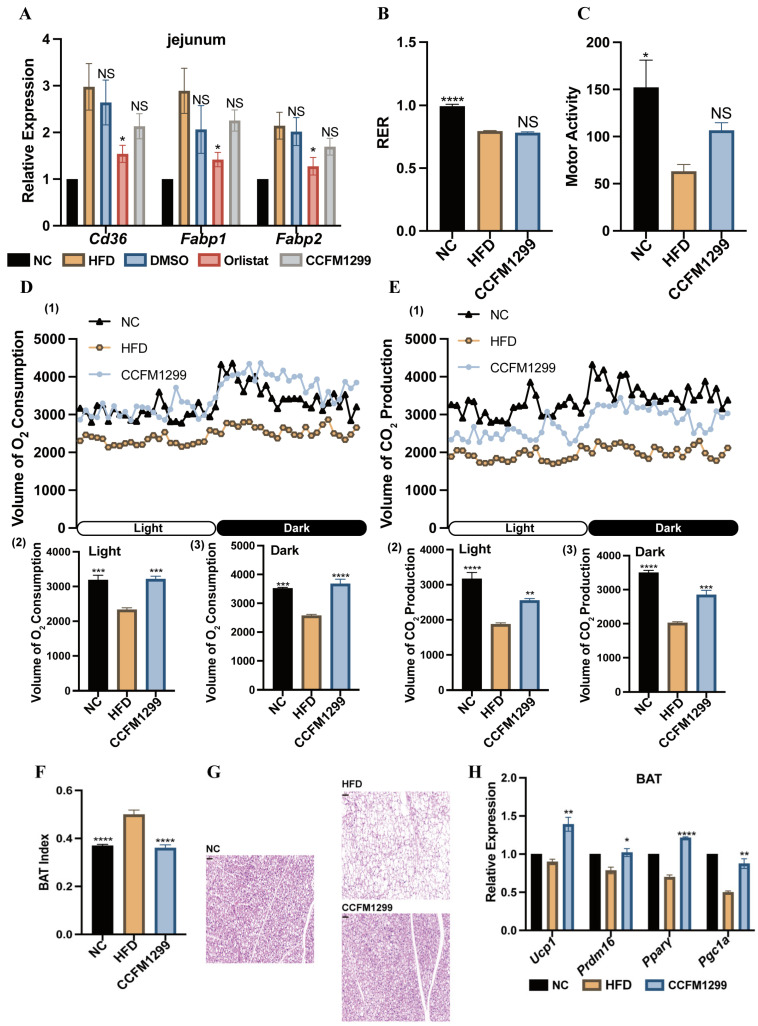
Effects of CCFM1299 on metabolism and the expression of thermogenesis-related genes in BAT: (**A**) the expression of *Cd36*, *Fabp1*, and *Fabp2* in jejunum; (**B**) RER of mice in NC, HFD, and CCFM1299 groups; (**C**) motor activity of mice in NC, HFD, and CCFM1299 groups; (**D**), (1) the O_2_ consumption curve of mice in a day; (2) the O_2_ consumption of mice in daytime (light); (3) the O2 consumption of mice in the night (dark); (**E**), (1) the CO_2_ production curve of mice in a day; (2) the CO_2_ production of mice in daytime (light); (3) the CO_2_ production of mice in the night (dark); (**F**) BAT index of mice in NC, HFD, and CCFM1299 groups; (**G**) representative histology of BAT in mice in NC, HFD, and CCFM1299 groups (scale bars: 50 µm; magnification: 200×); (**H**) the expression of Ucp1, Prdm16, *Pparγ*, and Pgc1a in BAT. Data are shown as means with standard error of the mean (SEM); One-way ANOVA followed by Dunnett’s test for multiple comparison was used for the significance analysis in (**A**–**F**), and in (**A**) the significance analysis was only performed between HFD, DMSO, orlistat, and CCFM1299 groups. (**A**–**F**) NS was used to indicate that there was no significant difference with HFD group. *, **, ***, and **** were used to indicate that the *p* values were less than 0.05, 0.01, 0.001, and 0.0001, respectively, compared with the HFD group. Student’s *t*-test was used for the significance analysis in (**H**).

As a lipase inhibitor, orlistat reduces body weight gain mainly by preventing dietary fat from hydrolyzing into fatty acids, which restricts the absorption of fat [21]. Given that strain CCFM1299 and orlistat both had anti-obesity effects, we investigated whether CCFM1299 had the potential to affect the lipid absorption of the small intestine as orlistat did. In order to test this, we detected the transcription levels of *Cd36*, *Fabp1*, and *Fabp2* in jejunum, which were associated with the transportation of fatty acids. The anti-obesity mechanism of CCFM1299 might differ from that of orlistat, as the transcription levels of these three genes decreased significantly only under the intervention of orlistat (Figure 3A). Increasing energy expenditure could help to reduce weight gain and eliminate obesity-related symptoms. We explored the influence of CCFM1299 on the energy expenditure of mice through a comprehensive monitoring system. An HFD made mice more inclined to use lipid as an energy substrate regardless of if CCFM1299 was orally given or not (Figure 3B). Without significantly changing the motor activity of mice (Figure 3C), the carbon dioxide (CO_2_) production and oxygen (O_2_) consumption of the CCFM1299 group were higher than those of the HFD group (Figure 3D,E). These suggested that the energy expenditure of mice was elevated by CCFM1299.

The thermogenesis of BAT is a way to promote energy expenditure. Under the intervention of CCFM1299, the BAT index of mice decreased (Figure 3F), along with the decreased accumulation of lipid droplets in BAT (Figure 3G). By detecting the transcription levels of thermogenic genes like *Ucp1*, *Pparγ*, *Pgc1α*, and *Prdm16* in BAT, we found that CCFM1299 intervention stimulated the transcription of these genes, which indicated that CCFM1299 had the potential to enhance thermogenesis in BAT under an HFD (Figure 3H).

### 3.4. CCFM1299 Had the Potential to Affect the Immune Homeostasis of eWAT

**Figure 4:**
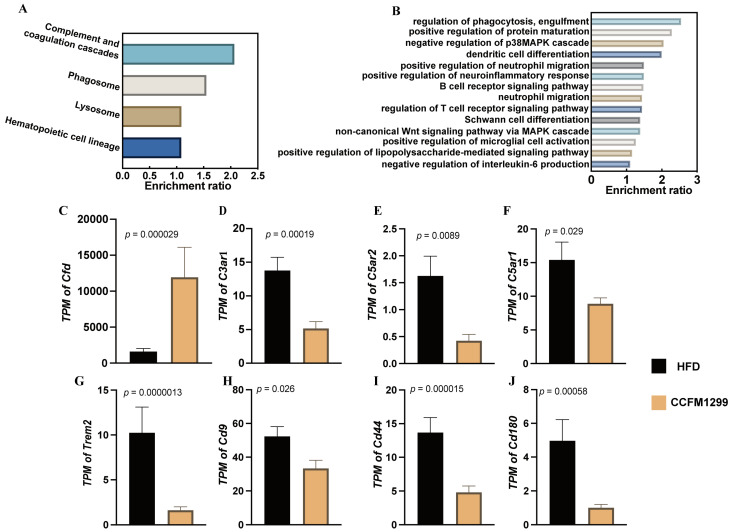
Effects of CCFM1299 on transcriptome of eWAT: (**A**) KEGG pathway enrichment analysis of DGs; (**B**) biological processes enriched via GO ontology; (**C–J**) *TPM* of differential genes involved in the enriched immune pathway.

WAT also played an important role in the perception of nutrition and the regulation of host metabolism. To explore the effect of CCFM1299 on eWAT, the global transcriptome of eWAT was analyzed. The differential genes (DGs) were selected using log_2_ (fold change) ≥1.5 or ≤–1.5, and *p* values < 0.05. GO ontology and KEGG pathway enrichment analysis were further performed to characterize the function of the DGs, and the pathways these genes participated in. Through the KEGG pathway enrichment analysis, DGs were enriched to the pathways that are shown in Figure 4A, and all the enriched pathways were involved in immune regulation. Similar to the KEGG pathway enrichment analysis, the biological processes (BPs) that were enriched via GO ontology were also related to the immune system (Figure 4B).

The complement system was proved to participate in the regulation of the immune system, mainly through modulating the engulfment ability and activation state of macrophages [22]. The transcription level of the complement factor D (CFD) gene was increased significantly under the intervention of CCFM1299, while the expressions of the complement receptor C3aR1, C5aR1, and C5aR2 genes were decreased (Figure 4C–F). In addition to this, we also found that the expressions of two genes (*Trem2* and *Cd9*) that encode the membrane markers in macrophages were downregulated by CCFM1299 (Figure 4G,H). The number of macrophages that expressed TREM2 and CD9 simultaneously were positively correlated with the generation of crown-like structures (CLS) and the intensive phagocytosis of lipid remnants in WAT [23]. CLS was usually seen in the eWAT of obese people and was associated with the occurrence of chronic inflammation [24]. This kind of macrophage also released the pro-inflammatory substance osteopontin (OPN) [25]. Excessive deposition of OPN in adipose tissue resulted in tissue fibrosis through activating CD44 (receptor for OPN), which finally aggravated the inflammatory response and impaired the metabolic plasticity [26]. In our study, the expression of the CD44 gene was downregulated by CCFM1299 (Figure 4I). All the above results indicated that strain CCFM1299 can affect the polarization of macrophages in eWAT, which might be associated with the complement system.

Moreover, the inflammation in WAT could be triggered by gastrointestinal antigens, in which lipopolysaccharide (LPS) was the most common antigen. Mechanically, the HFD might lead to intestinal barrier damage, and the pro-inflammatory LPS would migrate from the intestinal lumen to adipose tissue [27]. In our study, the expression of the *Cd180* gene (encoding receptor of LPS) was reduced under the administration of CCFM1299 (Figure 4J), and the BP, the positive regulation of the lipopolysaccharide-mediated signaling pathway, was also enriched through GO ontology analysis (Figure 4B). Thus, CCFM1299 might also ameliorate obesity-associated inflammation in eWAT partly by restoring the intestinal barrier.

### 3.5. CCFM1299 Affected the Composition of Bile Acid Metabolites

**Figure 5:**
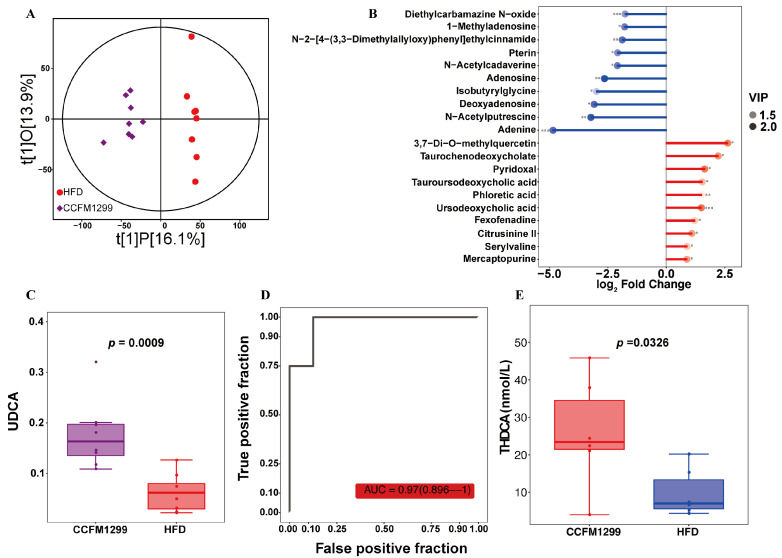
Effects of CCFM1299 on fecal and serum metabolites: (**A**) OPLS-DA of fecal metabolites between the HFD and CCFM1299 groups; (**B**) matchstick analysis of DMs between the CCFM1299 and HFD groups (the red matchstick indicated that the DMs were up-regulated in theCCFM1299 group, and the blue matchstick indicated that the DMs were downregulated in the CCFM1299 group); (**C**) the fecal level of UDCA showed in boxplot; (**D**) ROC of UDCA; (**E**) the serum concentration of THDCA. *, **, and ***were used to indicate that the *p* values were less than 0.05, 0.01, and 0.001, respectively.

Due to the fact that intestinal derivatives linked the symbionts and host, we finally examined the effects of CCFM1299 on host metabolites. First, non-targeted metabonomics was used to explore the effect of CCFM1299 on fecal metabolites. According to the orthogonal partial least squares discriminant analysis (OPLS-DA), the fecal metabolites of mice in the CCFM1299 group were apparently distinguished from those in the HFD group (Figure 5A). Then, we selected the differential metabolites between the HFD and CCFM1299 groups. The metabolites with VIP > 1, *p* < 0.05, and FC > 1.5 or FC < 0.5 were identified as the differential metabolites (DMs), which are shown in Figure 5B. In order to evaluate whether the anti-obesity effect of CCFM1299 could be attributed to the up-regulated DMs, we drew the receiver operating characteristic curve (ROC) of them and calculated the area under the curve (AUC). ROC is a commonly used method to evaluate the accuracy of diagnoses in clinical medicine and epidemiological research, and it could also be applied to appraising the correlation between endogenous substances and diseases (AUC > 0.9, with high correlation) [28]. Under the intervention of CCFM1299, the fecal level of UDCA was increased significantly, and it was also extremely related to the improvement of obesity (Figure 5C,D, AUC = 0.97). Furthermore, the composition of bile acids in serum was detected by targeted metabonomics, and we found that CCFM1299 intervention significantly increased the concentration of THDCA in serum (Figure 5E). Therefore, the CCFM1299 administration might alleviate obesity by influencing the bile acid metabolism in mice.

## 4. Discussion

A growing number of studies show that intestinal symbionts can alleviate obesity and related diseases [29]. Compared with pharmacotherapy, the supplementation of these symbiotic bacteria could reduce obesity without producing obvious side effects. Instead, it might prevent diarrhea by regulating intestinal microecology [30]. *Clostridium butyricum,* one of the intestinal symbionts, had beneficial effects on a range of diseases, such as obesity, inflammatory bowel disease, and neurodegenerative diseases [31,32]. The *Clostridium butyricum* strain CCFM1299 with anti-obesity effects was screened in our previous study, and the potential of this strain to alleviate obesity was once again confirmed in this study, mainly manifested in inhibiting weight gain, lowering FBG and lipid level, and preventing liver damage. Moreover, the possible ways strain CCFM1299 alleviates obesity have been explored, and the effects that it brought to the intestinal and serum metabolites have also been discussed in this study.

In essence, the imbalance between energy absorption and consumption leads to obesity. We first explored the effects of CCFM1299 and orlistat on lipid absorption by detecting the expression of the *Cd36*, *Fabp1*, and *Fabp2* genes involved in fatty acid transportation in the intestine. As expected, orlistat reduced the expression of *Cd36*, *Fabp1*, and *Fabp2*. However, CCFM1299 administration did not, apparently, alter the expression of the above genes, which indicated that strain CCFM1299 might not alleviate obesity via affecting lipid absorption in the intestine. Even though preventing dietary lipid from absorbing was a way to relieve obesity, the adverse gastrointestinal reactions caused by orlistat were related to its inhibition of fat absorption [33]. This study has shown that in addition to fatty acid transportation, CD36 was also involved in phagocytosis, antigen presentation, and the clearance of apoptosis cells, which played an essential role in the maintenance of intestinal homeostasis and the integrity of the intestinal barrier [34]. Thus, we suspected that oral administration of CCFM1299 was less likely to produce adverse gastrointestinal reactions than orlistat.

Afterwards, the metabolism of mice was monitored. We found that strain CCFM1299 increased the CO_2_ production and O_2_ consumption of mice without changing the motor activity significantly, which indicated that the oral administration of CCFM1299 reduced obesity partly via boosting the metabolism and promoting energy consumption in mice. BAT is a kind of adipose tissue consuming energy in the form of non-shivering heat production [35]. Some studies pointed out that BAT was a target to prevent the development of obesity, as enhancing its thermogenic activity could increase insulin sensitivity and ameliorate lipid metabolism [36]. Therefore, we suspected that strain CCFM1299 might improve energy consumption by affecting the thermogenic process of BAT. In order to verify this, we explored the expression of the genes related to the thermogenesis of BAT. The results showed that strain CCFM1299 increased the transcription level of key thermogenic genes (*Ucp1*, *Pparγ*, *Pgc1α*, and *Prdm16*). In addition, the whitening of BAT under an HFD was also prevented by CCFM1299. The morphological differences of BAT between groups with or without CCFM1299 intervention demonstrated that the lipolysis of BAT might be stimulated by CCFM1299. Lipolysis is the beginning of thermogenesis since it provides fatty acid as the thermogenic substrates. To sum up, CCFM1299 could promote the utilization of lipid by BAT and activate the thermogenesis process, which eventually helps to reinforce whole-body energy expenditure.

In addition to BAT, WAT also plays an important role in the pathological process of obesity. WAT is not only an organ for energy storage, but also participates in the maintenance of energy and immune homeostasis of the host. Through transcriptome analysis, we found that CCFM1299 mainly affected the expression of genes related to immune regulation in eWAT. In obese individuals, macrophages were recruited into WAT, which surrounded the apoptotic adipocytes and engulfed them. Although this process could prevent toxic lipids released from necrotic adipocytes, it would lead to the excessive accumulation of lipids in macrophages, which eventually turn macrophages into pro-inflammatory phenotypes. CD9 and Trem2 were the plasma membrane markers of this pro-inflammatory, lipid-associated macrophage [37], and the lean mice receiving the CD9 + macrophages from obese mice displayed severe metabolic disorders [38], which indicated the causality between this macrophage subtype and obesity.

The lipid intake and pro-inflammatory polarization of macrophages could be inhibited by the CFD, a serine protease in complement systems, which was essential in the immune homeostasis in WAT [39]. Under the intervention of CCFM1299, we found that the increased expression of the CFD gene was accompanied by the decreased transcription levels of the *Cd9* and *Trem2* genes. These indicated that CCFM1299 might affect the polarization of macrophages by regulating the content of CFD in eWAT. Different from the CFD, the increased expression of complement receptors C3aRs and C5aRs not only caused macrophage infiltration in adipose tissue [40], but also enhanced the LPS-induced inflammatory reaction in adipose tissue [41]. Consistent with the above-mentioned studies, we found that oral administration of CCFM1299 not only reduced the expression levels of the C3aR1, *C5aR1*, and *C5aR2* genes, but also reduced the expression levels of *Cd180* (receptor for LPS). Overall, strain CCFM1299 might regulate the immune homeostasis of eWAT by affecting the complement system, through which it restrains the development of obesity.

In our previous study, we found that the *Clostridium butyricum* strain CCFM1299 fails to restore the concentration of butyrate in cecum. Thus, the effects of strain CCFM1299 on fecal and serum metabolites were explored in this study through targeted and non-targeted metabonomics. Based on non-targeted metabonomics, we first revealed the effects of strain CCFM1299 on fecal metabolites. Under the intervention of CCFM1299, the concentrations of UDCA, TCDCA, and TUDCA were increased significantly in feces, among which UDCA had the highest diagnostic accuracy. Research claims that oral administration of UDCA could inhibit the inflammation of eWAT, and the fecal level of UDCA was negatively related to the metabolic disorders and intestinal permeability of mice fed with an HFD [42]. The alteration of fecal metabolites was a cue that bile acid metabolism might be influenced by CCFM1299. Thus, the effect of strain CCFM1299 on serum bile acids was investigated using targeted metabonomics. The results showed that strain CCFM1299 significantly increased the content of THDCA in serum.

The increase in serum THDCA level was related to the relief of liver fibrosis and the decreased risk of diabetes [43], which showed that THDCA had the potential to amend the symptoms of obesity and related diseases. In addition, THDCA was also able to restrict inflammatory effects by promoting the polarization of Treg cells and regulating Th1/Th2 cell balance [44]. THDCA belongs to hycholic acids (HCAs), which can be classified into 6α-hydroxylated bile acids. This kind of bile acid promoted the production of GLP-1 in the intestine via activating TGR5 and inhibiting FXR, which helped to regulate blood glucose homeostasis and decrease the morbidity of obesity and complications [45]. Notably, the aforementioned UDCA and CDCA were the primary bile acids in murine, which could be metabolized into HCAs by specific bacteria expressed as 7- dehydroxylase (baiJ) [46]. As *Clostridium* was the main genera that expressed bai [47], the strain CCFM1299 might be responsible for the transformation from UDCA or CDCA to THDCA. However, this hypothesis needs to be verified in vitro.

## 5. Conclusions

Overall, the results obtained from this research revealed that the *Clostridium butyricum* strain CCFM1299 prevented the development of obesity via affecting the expression of thermogenic genes in BAT, which has not been reported yet. In addition, CCFM1299 also modulated the expression of the genes associated with immune homeostasis in eWAT. Although the immune regulation of *Clostridium butyricum* on adipose tissue has been reported, our results revealed that such regulation might be associated with the complement system in eWAT. In terms of metabolites, CCFM1299 increased the fecal concentration of UDCA and the serum level of THDCA. It has been known that specific bile acids could regulate thermogenesis and immune homeostasis in the host. Therefore, our research provided a new insight into the mechanism of *Clostridium butyricum* strain on relieving obesity and laid a theoretical foundation for developing anti-obesity *Clostridium butyricum* preparation.

## Data Availability

RNA-seq data were published on NCBI and the accession number is PRJNAA992647.
